# Proceedings of the Homœopathic Medical Society, of Ohio

**Published:** 1884-11

**Authors:** 


PROCEEDINGS OF THE TWENTIETH ANNUAL SESSION OF THE HOMŒOPATHIC MEDICAL SOCIETY OF OHIO, held at Cleveland, May 13th, 1884.

Through the kindness of Dr. Beebe, the Secretary, we are again presented with a copy of the proceedings of the large and able Society of Ohioan homœopathists. The volume gives evidence of a prosperous medical society, is interesting, but, like all our medical works, shows neglect of therapeutic work.

